# Causal Effects of Plasma Metabolites on Leukemia: A Mendelian Randomization Study

**DOI:** 10.3390/metabo15110719

**Published:** 2025-11-03

**Authors:** Chang Huang, Yuchen Li, Mengjie Li, Xu Ye, Tong Wang, Nannan Liu, Xinyi Meng, Yu Gao, Xinhui Wang

**Affiliations:** 1 Zhejiang Hospital, Department of Hematology, Hangzhou 310000, China; 2 School of Public Health, Institute of Nutrition and Food Safety, Zhejiang University School of Medicine, Hangzhou 310058, China

**Keywords:** mendelian randomization, plasma metabolites, leukemia, causal inference, pharmacologic leads

## Abstract

**Background:** Leukemia comprises heterogeneous hematologic malignancies, and whether circulating metabolites contribute causally to subtype-specific risk remains uncertain. **Objectives:** The aim of this study was to assess the causal effects of plasma metabolites for acute myeloid leukemia (AML), chronic myeloid leukemia (CML), acute lymphoblastic leukemia (ALL), and chronic lymphocytic leukemia (CLL). **Methods:** A two-sample Mendelian randomization (MR) using summary-level genome-wide association study statistics was conducted. For each metabolite, a single variant showing the strongest association with the metabolite that had the largest variance explained (R^2^) among the independent genome-wide significant (*p* < 5 × 10^−8^) SNPs assigned to effector genes was selected as sentinel. Multiple comparisons using Bonferroni correction (0.05/83 = 6.02 × 10^−4^) were applied to minimize the risk of obtaining false positive results. **Results:** Totally, 83 metabolites and metabolite ratios were analyzed for AML, CML, ALL, and CLL. Lithocholate sulfate (1), instrumented by the rs10425975 variant in the SULT2A1 gene, was significantly associated with an increased risk of CLL (OR = 2.19; 95% CI: 1.45–3.31; *p* = 2 × 10^−4^). An additional 17 metabolite-leukemia associations showed suggestive evidence of significance. Approximately 300 drug entries linked to candidate metabolites were curated to provide a basis for mechanistic follow-up. **Conclusions:** Our MR result supports a causal link between higher genetically proxied lithocholate sulfate (1) and increased CLL risk. The discovery of these “metabolite-gene-drug” relationships suggests a central role in leukemia pathogenesis and warrants further functional investigation for their therapeutic potential.

## 1. Introduction

Leukemia comprises biologically and clinically heterogeneous hematologic malignancies defined by lineage (myeloid vs. lymphoid) and clinical tempo (acute vs. chronic), including acute myeloid leukemia (AML), chronic myeloid leukemia (CML), acute lymphoblastic leukemia (ALL), and chronic lymphocytic leukemia (CLL) [1]. These subtypes differ markedly in cell of origin, molecular profiles, clinical outcomes, and treatment response, and the overall disease burden is rising in many regions [2]. In parallel, the International Consensus Classification (ICC) provides a harmonized, evidence-based framework that refines diagnostic boundaries and prognostic groupings across myeloid neoplasms and acute leukemias [3]. Collectively, these frameworks underscore that subtype-specific etiology is the biologically appropriate unit for epidemiology and translational research, and they motivate analyses that respect recognized genomic and clinical heterogeneity [1,3,4].

Metabolic dysregulation is a hallmark of cancer and affects lineage commitment, genome maintenance, and tumor–immune interactions. Work in myeloid malignancies illustrates how metabolite changes can act as proximal drivers of leukemogenesis. Mutant IDH1 and IDH2 generate 2-hydroxyglutarate, disrupt TET2 function, enforce DNA hypermethylation, and impair differentiation in acute myeloid leukemia, showing that metabolic alterations can be causal rather than merely correlative [5].

Conventional observational studies have long reported metabolic abnormalities in leukemia across amino acid, lipid, and organic-acid pathways, but such evidence is vulnerable to confounding and reverse causation. Cross-sectional or cohort correlations cannot by themselves establish whether metabolic changes are causes of disease or consequences of it. Mendelian randomization uses germline variants strongly associated with the exposure, namely plasma metabolite levels, as instrumental variables. Under its core assumptions, Mendelian randomization (MR) reduces confounding and mitigates reverse causation bias, providing complementary causal evidence to randomized trials [6]; The STROBE-MR guidelines further standardize the design and reporting of MR studies [7,8].

Recent metabolite GWAS have created a robust “instrument library” for MR. For example, a 2023 Nature Genetics study reported association signals for 1091 plasma metabolites and 309 biologically constrained ratios, mapping the genetic architecture of the plasma metabolome and linking it to complex traits. Earlier work had already charted genetic influences on more than 400 blood metabolites [9,10]. These public resources make two-sample MR with metabolite exposures feasible by harmonizing genome-wide significant metabolite instruments with leukemia GWAS outcomes to estimate causal effects. 

Despite progress, an important knowledge gap remains: while metabolite genetics has been mapped broadly and MR has implicated metabolites in diverse complex traits, the causal relevance of specific plasma metabolites for leukemia subtypes has not been systematically evaluated at scale using a harmonized, single-sentinel framework [9]. Addressing this gap is timely given etiologic heterogeneity recognized by modern disease classifications and the emergence of metabolism-targeted strategies in myeloid malignancies (e.g., exploiting differentiation checkpoints or mitochondrial dependencies) that make mechanistic follow-up of causal leads actionable [1].

## 2. Method

### 2.1. Data Sources and Study Design

A two-sample Mendelian randomization was conducted to evaluate the causal effects of human plasma metabolites on leukemia. Plasma metabolite summary statistics came from the Canadian Longitudinal Study on Aging (CLSA) cohort with data deposited in the GWAS Catalog under accession ranges GCST90199621–GCST90201020 for European ancestry [9]. The GWAS for metabolites and their ratios included 8299 individuals. Summary statistics for leukemia were taken from the FinnGen R12 and UK Biobank meta-analysis resource [11]. The case and control counts were as follows: acute myeloid leukemia 731 and 793,587; chronic myeloid leukemia 474 and 793,588; acute lymphoblastic leukemia 313 and 706,277; chronic lymphocytic leukemia 1585 and 793,582.

**Figure 1:**
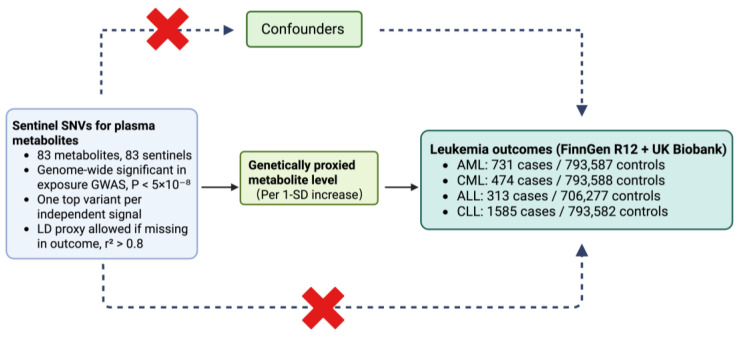
Two-sample Mendelian randomization framework for metabolite–leukemia analyses. Sentinel SNVs were selected from the exposure GWAS at genome-wide significance (*p* < 5 × 10^−8^), retaining one top variant per independent signal across 83 metabolites; if a sentinel was absent in an outcome file, a matched-ancestry LD proxy with r^2^ > 0.8 was used. The associations of these instrumental SNVs with all metabolites and outcomes were harmonized to the same effect allele, and causal effects were estimated per 1-SD increase in genetically predicted metabolite levels. Outcomes were from FinnGen R12 and UK Biobank: AML 731/793,587; CML 474/793,588; ALL 313/706,277; CLL 1585/793,582. ALL, acute lymphoblastic leukemia; AML, acute myeloid leukemia; CLL, chronic lymphocytic leukemia; CML, chronic myeloid leukemia; LD, linkage disequilibrium; SD, standard deviation; SNV, single-nucleotide variant.

Each metabolite was treated as an exposure and each leukemia subtype as a separate outcome. Because clinical and genetic architectures differ by subtype, all analyses were performed independently for each outcome and results were reported in parallel. Exposure and outcome ancestries were aligned as closely as possible according to the source studies. Outcome effects are on the log-odds scale and are presented as odds ratios after exponentiation for interpretability. The single-nucleotide variations (SNVs) used to demonstrate causal effects in the MR analysis must satisfy three key assumptions: Firstly, the IVs must be closely related to the exposure; Secondly, the IVs are not related to any confounders of the risk factor-outcome association; Thirdly, the IVs do not affect the outcome through any pathway other than the exposure of interest [12].The fundamental assumptions of MR are depicted in Figure 1. A consolidated overview of exposure and outcome GWAS sources, including consortium/study, ancestry, sample sizes by subtype (AML, CML, ALL, CLL), data access links, and primary references, is tabulated for transparency and replication (Supplementary Table S1). Pharmacologic annotations linking metabolites to drugs were obtained from DrugBank.

### 2.2. Instrument Selection and Data Harmonization

Instrumental variables were defined using a single-sentinel strategy for the exposure. In this study, the sentinel was defined as the SNV showing the strongest association with the metabolite that had the largest variance explained (R^2^) among the independent genome-wide significant (*p* < 5 × 10^−8^) SNPs assigned to effector genes. To minimize pleiotropic effects, we excluded the FADS gene region, which is associated with multiple metabolites, as well as the major histocompatibility complex region on chromosome 6. This sentinel instrumental variable selection strategy enhances the representativeness, interpretability, and therapeutic relevance of the analysis. For metabolites with multiple independent signals, only the top sentinel was retained for the primary analysis, yielding a one-to-one mapping of metabolites to instruments. The final instrument list comprised 83 unique metabolites and 83 corresponding sentinels.

When a sentinel was missing in an outcome file, an LD proxy was permitted under a predefined rule. Proxies were searched within a matched-ancestry reference with r^2^ > 0.8; when multiple candidates were available, the proxy with the highest r^2^ was selected, and the proxy indicator with its r^2^ value was carried forward into the analysis dataset. Effect alleles and allele frequencies were then harmonized between exposure and outcome, including sign changes when required by strand orientation.

For each outcome, exposure and outcome tables were formatted to comply with “TwoSampleMR” and harmonized to the same effect allele. Palindromic variants with adequate allele-frequency information were resolved; unresolved strand conflicts and non-A/T/C/G alleles were excluded. Instruments were required to meet the exposure-side genome-wide significance threshold and to have complete fields for effect size, standard error, effect and other allele, and allele frequency. Independence had already been enforced by the single-sentinel rule; accordingly, no additional clumping was performed at the MR stage.

### 2.3. MR Estimation, Pleiotropy Control, and Statistical Inference

Because each metabolite contributed a single instrument, the primary estimator was the Wald ratio as implemented in TwoSampleMR. For binary outcomes we report the odds ratio per one standard-deviation higher genetically predicted metabolite level together with ninety-five percent confidence intervals.

To quantify instrument strength, we computed the *R*^2^ for each sentinel from the exposure-side effect size and allele frequency, and then derived the first-stage F statistic using:

$$F=\frac{R^{2}/k}{\left(1-R^{2})(n-k-1\right)}$$

where *k* = 1 in the single-instrument setting and *n* is the exposure GWAS sample size for that metabolite. When the exposure was SD-scaled, *R*^2^ was obtained using standard single-variant calculations. These metrics accompany the MR estimates and were not used for additional filtering beyond the exposure-side genome-wide significance threshold. We also summarized post hoc power at prespecified odds-ratio effect sizes of 0.90, 1.20, and 1.50 while accounting for the case–control ratio of each outcome.

To limit horizontal pleiotropy, we performed a programmatic screen of every sentinel or selected proxy against the GWAS Catalog using the gwasrapidd (version 0.99.18) in R (version 4.5.1; R Foundation for Statistical Computing). The catalog queries were run in June 2025. We flagged genome-wide significant associations in domains that could constitute alternative pathways to leukemia risk, including neoplastic, immune, hematologic, and cardiometabolic traits. Variants flagged by this screen were compiled into a drop list, and the corresponding rsIDs were removed from the MR input. In addition, if a sentinel or proxy showed genome-wide significance for the target leukemia outcome itself, that variant was removed before MR estimation. We retained the catalog annotations for the remaining instruments to aid interpretation in the results. Prior genome-wide associations for each sentinel (and eligible proxies) were retrieved from the GWAS Catalog to flag potential alternative biological pathways and to inform variant exclusion; detailed cross-references are provided in Supplementary Table S2.

For multiplicity control all *p* values are two-sided, and our primary decision rule is Bonferroni within outcome. With eighty-three metabolites per outcome the significance threshold equals 0.05 divided by eighty-three (0.05/83), which is 6.02 × 10^−4^. Findings that meet the relevant Bonferroni threshold are described as statistically significant. Findings with a *p* value less than 0.05 that do not meet the Bonferroni threshold are described as having suggestive significance and are treated as exploratory rather than primary evidence.

All analyses were conducted in R (version 4.5.1; the R Foundation for Statistical Computing). Data were analyzed using the TwoSampleMR (version 0.6.22) and MendelianRandomization (version 0.10.0) packages. The core packages were TwoSampleMR for data formatting, allele harmonization, and Wald ratio estimation; MendelianRandomization for complementary utilities; and data.table (version 1.17.0), dplyr (version 1.1.4), tidyr (version 1.3.1), reshape2 (version 1.4.4), ggplot2 (version 4.0.0), forestplot (version 3.1.7), plotly (version 4.11.0), htmlwidgets (version 1.6.4), and webshot (version 0.5.5) for data handling, visualization, and export. The scripted workflow exports harmonized exposure–outcome tables, MR results with odds ratios and confidence intervals and method labels and *p* values, and instrument-strength summaries including variance explained, F statistic, and exposure sample size to support reproducibility.

## 3. Results

### 3.1. Metabolite-Associated SNVs

At first, a total of 171 metabolite-associated SNVs were selected. A total of 103 metabolite-associated SNVs remained after excluding non-canonical variants. Based on rsIDs, 102 SNVs were identified in the outcome GWAS to refine the proxy genes. Furthermore, based on phenotypic context, 20 SNVs associated with phenotypes related to major diseases such as tumors and the immune system were removed, resulting in a total of 83 strongly metabolite-associated SNVs (Supplementary Table S3). R^2^ and F statistics for each instrument are reported in Supplementary Table S4. Consistent with the conventional MR rule that instrument strength should exceed F greater than 10, all sentinel instruments met this requirement; after ranking by F, the smallest value was 36.5 and the largest was 4013, indicating high overall instrument quality (Supplementary Table S4).

### 3.2. Relationship Between Metabolites and Leukemia

**Figure 2:**
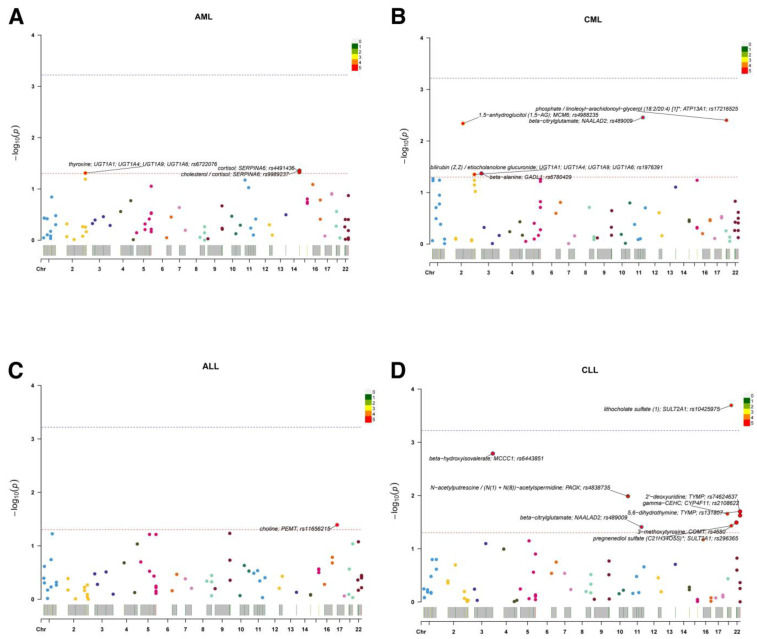
Single-sentinel MR scans of plasma metabolites by leukemia subtype. (**A**) AML, (**B**) CML, (**C**) ALL, and (**D**) CLL. Each point is one metabolite–outcome test using the metabolite’s sentinel SNV; the x-axis places the sentinel by chromosome, and the y-axis shows −log_10_(P). Labels mark the top signals (metabolite; annotated gene; sentinel rsID). The upper dashed line denotes the significance (α = 0.05/83); the lower dashed line denotes suggestive significance (*p* = 0.05). A significant association is observed for lithocholate sulfate (1) in panel D (CLL); other panels display suggestive signals only. An asterisk indicates that metabolite unit: 1 s.d. of log-normalized values and metabolite ratio unit: 1 s.d. of inverse rank normalized values. ALL, acute lymphoblastic leukemia; AML, acute myeloid leukemia; CLL, chronic lymphocytic leukemia; CML, chronic myeloid leukemia; MR, Mendelian randomization; SNV, single-nucleotide variant.

Totally, 83 metabolites and metabolite ratios were analyzed for MR association with AML, CML, ALL, and CLL. The Wald ratio method was used to directly use the effect size estimate of a single SNV to infer the causal relationship between exposure and outcome. After correcting for multiple comparisons, only lithocholate sulfate (1) (rs10425975; SULT2A1) showed a positive association with CLL (OR = 2.19; 95% CI: 1.45–3.31; *p* = 2 × 10^−4^). Other metabolite–leukemia pairs were at suggestive level, including cortisol (OR = 2.83; 95% CI: 1.01–7.93; *p* < 0.05) was positively associated with AML, cholesterol/cortisol (OR = 0.35; 95% CI: 0.13–0.97; *p* < 0.05) and thyroxine (OR = 0.34; 95% CI: 0.12–1.00; *p* < 0.05) were negatively associated with AML, phosphate/linoleoyl-arachidonoyl-glycerol (18:2/20:4) [1]* (OR = 6.32; 95% CI: 1.80–22.15; *p* < 0.01) and 1,5-anhydroglucitol (1,5-AG) (OR = 4.99; 95% CI: 1.64–15.14; *p* < 0.01) were positively associated with CML, beta-citrylglutamate (OR = 0.63; 95% CI: 0.46–0.86; *p* < 0.01), beta-alanine (OR = 0.50; 95% CI: 0.26–0.98; *p* < 0.05), and bilirubin (Z,Z)/etiocholanolone glucuronide (OR = 0.67; 95% CI: 0.46–0.99; *p* < 0.05) were negatively associated with CML, choline (OR = 4.14; 95% CI: 1.06–16.10; *p* < 0.05) was positively associated with ALL, beta-hydroxyisovalerate (OR = 2.55; 95% CI: 1.42–4.57; *p* < 0.01), 2′-deoxyuridine (OR = 1.67; 95% CI: 1.07–2.59; *p* < 0.05), 3-methoxytyrosine (OR = 2.24; 95% CI: 1.07–4.70; *p* < 0.05), and pregnenediol sulfate (C21H34O5S)* (OR = 2.13; 95% CI: 1.05–4.33; *p* < 0.05) were positively associated with CLL, N-acetylputrescine/(N(1) + N(8))-acetylspermidine (OR = 0.55; 95% CI: 0.35–0.87; *p* < 0.05), 5,6-dihydrothymine (OR = 0.46; 95% CI: 0.24–0.88; *p* < 0.05), gamma-CEHC (OR = 0.73; 95% CI: 0.56–0.96; *p* < 0.05), and beta-citrylglutamate (OR = 0.83; 95% CI: 0.70–0.99; *p* < 0.05) were negatively associated with CLL (Supplementary Table S5, Figure 2).

**Figure 3:**
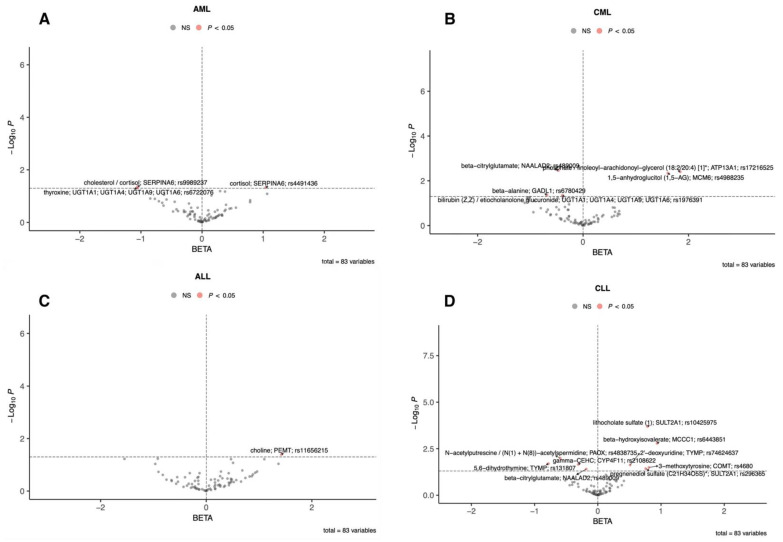
Single-sentinel MR volcano plots of plasma metabolites by leukemia subtype. (**A**) AML, (**B**) CML, (**C**) ALL, and (**D**) CLL. Each point is one metabolite–outcome test using that metabolite’s sentinel SNV with a Wald-ratio estimate. The x-axis is the MR β (log-odds per 1-SD higher genetically proxied metabolite level): β > 0 indicates higher risk (risk-increasing), β < 0 indicates lower risk (risk-decreasing). The y-axis is “−log_10_(P)”. The vertical dashed line marks “β = 0”; the horizontal dashed line marks the *p* = 0.05 suggestive threshold (red points: *p* < 0.05; gray: not significant). Labels mark top signals (metabolite; annotated gene; sentinel rsID). An asterisk indicates that metabolite unit: 1 s.d. of log-normalized values and metabolite ratio unit: 1 s.d. of inverse rank normalized values. ALL, acute lymphoblastic leukemia; AML, acute myeloid leukemia; CLL, chronic lymphocytic leukemia; CML, chronic myeloid leukemia; MR, Mendelian randomization; SNV, single-nucleotide variant.

**Figure 4:**
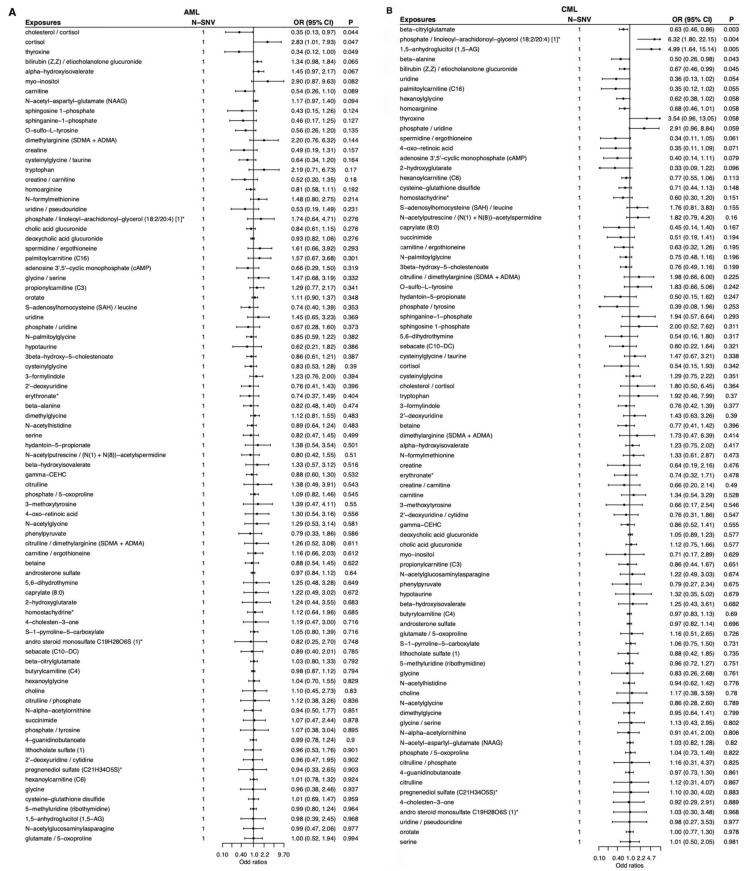

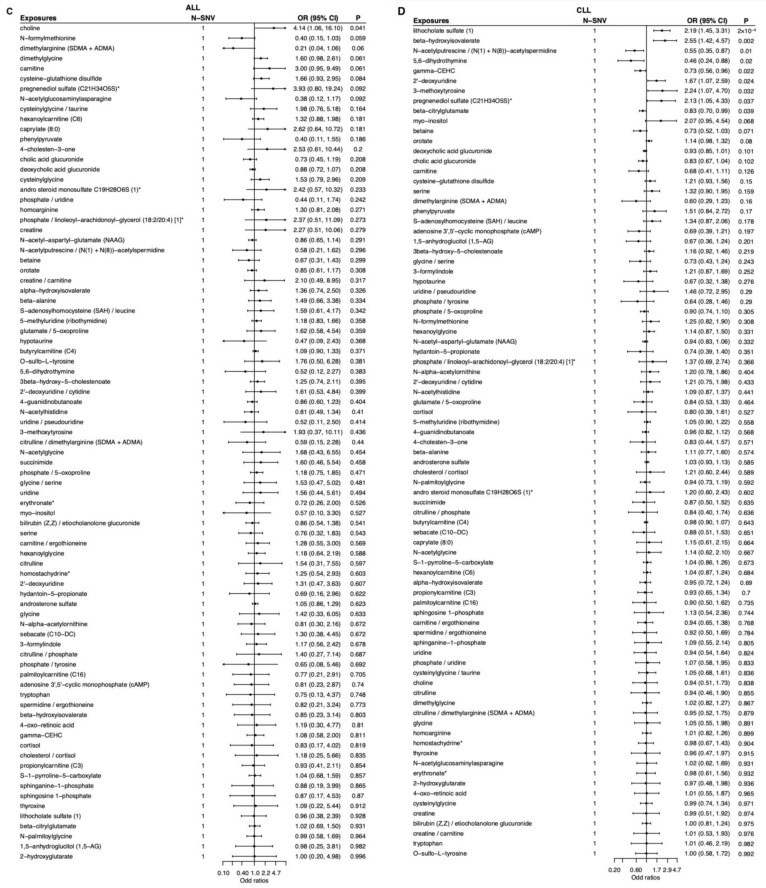
Single-sentinel MR forest plots of plasma metabolites by leukemia subtype. (**A**) AML, (**B**) CML, (**C**) ALL, and (**D**) CLL. Each row is one metabolite–outcome test using that metabolite’s sentinel SNV and the Wald-ratio estimator. Points show odds ratios (OR) per 1-SD higher genetically proxied metabolite level; horizontal bars are 95% CIs. The vertical dashed line marks the null (OR = 1). Values < 1 indicate inverse (protective-direction) associations; values > 1 indicate positive (risk-direction) associations. The “N-SNV” column (=1 for all rows) indicates that each estimate is based on a single instrument. An asterisk indicates that metabolite unit: 1 s.d. of log-normalized values and metabolite ratio unit: 1 s.d. of inverse rank normalized values. ALL, acute lymphoblastic leukemia; AML, acute myeloid leukemia; CLL, chronic lymphocytic leukemia; CML, chronic myeloid leukemia; MR, Mendelian randomization; SNV, single-nucleotide variant.

To visualize both direction and strength of effects, single-sentinel MR volcano plots (β on the x-axis, −log_10_P on the y-axis) are shown in Figure 3, and corresponding forest plots reporting odds ratios per 1-SD higher genetically proxied metabolite level with 95% CIs are shown in Figure 4. Single-sentinel, per-metabolite MR estimates on an interpretable scale (odds ratio per 1-SD higher genetically proxied exposure), with Beta, SE, 95% CI, P, and method labels, are provided by subtype in the supplement (Supplementary Tables S6–S9 for AML, CML, ALL, and CLL, respectively). To aid interpretation of null and suggestive findings, statistical power was evaluated at prespecified odds ratios of 0.90, 1.20, and 1.50 for each leukemia subtype; subtype-specific summaries are provided in Supplementary Tables S10–S13.

### 3.3. Metabolites and Drugs

Only one metabolite showed significant differences in leukemia activity, and seven metabolites were associated with it. Seventeen metabolites showed suggestive statistical differences in leukemia activity (Supplementary Table S5). Based on phenotypic changes in knockout mice, human Mendelian traits and disease, and pharmacological relevance of 94 effector genes (Supplementary Table S14), 300 drugs were associated with it (Supplementary Table S14). Beyond significance, pharmacologic context was profiled for the CLL-significant metabolite. Using DrugBank, Table 1 lists clinical drugs that interact with its assigned effector enzyme SULT2A1—abiraterone, ibrexafungerp, methyldopa, palbociclib, pindolol, prasterone, tamoxifen, and terbutaline, each annotated as a substrate of SULT2A1 rather than an antagonist, agonist, inhibitor, or inducer. This substrate-only pattern points to biotransformation by the effector gene product rather than direct receptor modulation of the metabolite.

## 4. Discussion

In this study, we performed a two-sample MR analysis using a single-sentinel strategy and summary statistics from large-scale plasma metabolome and subtype-specific leukemia GWAS datasets to evaluate the causal effects of circulating metabolites on leukemia risk. The design and reporting of this study were conducted in accordance with the STROBE-MR guidelines (Supplementary Table S15). This approach addresses the current lack of subtype-resolved causal evidence. Using a uniform MR framework, we tested whether genetically proxied metabolite levels influence the risks of AML, CML, ALL, and CLL. Leveraging comprehensive metabolite and leukemia GWAS resources, we estimated subtype-specific causal effects on interpretable risk scales while applying consistent instrument definitions. Finally, by integrating these causal estimates with curated biological resources such as DrugBank and the GWAS Catalog, we provide a transparent, subtype-resolved landscape of metabolite–leukemia relationships that highlights prioritized metabolic pathways for replication and mechanistic exploration.

Metabolites are intimately linked to leukemogenesis and disease progression, and numerous studies have demonstrated that metabolic pathways exert key roles in leukemia biology. For example, IDH1/2 mutations alter enzyme function and catalyze the conversion of α-ketoglutarate to the oncometabolite R-2-hydroxyglutarate [13,14]. IDH1/IDH2 mutations increase the risk of transformation from myelodysplastic syndrome (MDS) to high-risk AML and are associated with poor prognosis in AML, underscoring their role in disease progression [15,16]. In MLL-rearranged AML, leukemia stem cells (LSCs) are highly dependent on the purine biosynthesis pathway; CRISPR screens show that purine biosynthetic genes are selectively upregulated in LSCs, and MYC-driven purine metabolism maintains LSC stemness and differentiation arrest [17]. Other work indicates that decitabine can enhance CD36-mediated immunosuppression in leukemia cells, leading to chemotherapy resistance, whereas statins may improve decitabine efficacy by reducing lipoproteins transported via CD36 and thereby alleviating immunosuppression [18]. In addition, newly diagnosed CML patients exhibit elevated plasma lactate, consistent with augmented glycolysis; lactate levels decrease after tyrosine kinase inhibitor therapy, indicating that metabolic reprogramming tracks with disease activity [19].

Studies have also shown that leukemia cells exist a “metabolic addiction” to key metabolic pathways. In AML, LSCs are highly dependent on amino acid energy and mitochondrial oxidative phosphorylation (OXPHOS). The combination of venetoclax and azacitidine can selectively target LSCs by reducing amino acid uptake and depleting OXPHOS [20,21]. Resistance along this axis is frequently accompanied by upregulated fatty-acid oxidation (FAO); FAO inhibition reduces the fitness of stem/progenitor-like leukemic populations in vitro and significantly increases sensitivity to apoptosis inducers and chemotherapy [22,23]. Separately, dihydroorotate dehydrogenase (DHODH)—the rate-limiting enzyme in de novo pyrimidine synthesis—acts as a metabolic checkpoint for myeloid differentiation in multiple AML models: selective DHODH inhibition induces differentiation and reduces leukemia-initiating activity, and the effect is fully rescued by exogenous uridine, indicating that the mechanism reflects uridine monophosphate supply restriction rather than off-target effects. In vivo, DHODH inhibitors (e.g., brequinar) reduce tumor burden and prolong survival at tolerable dosing schedules, suggesting a therapeutic window [24]. In addition, MTHFD2, a mitochondrial one-carbon metabolism enzyme, is consistently upregulated across cancers and functionally required in AML [25,26]. Pharmacologic or genetic inhibition of MTHFD2 markedly suppresses leukemia progression both in vitro and in vivo by constraining one-carbon metabolism, leading to deoxynucleotide and thymidylate depletion, replication stress, S-phase arrest, and subsequent apoptotic cell death, thereby delineating a therapeutically exploitable metabolic vulnerability.

Metabolites play a crucial role in many aspects of leukemia, including the pathogenesis and progression of the disease. However, the large number of metabolites makes traditional research methods inefficient, labor-intensive, and subject to significant confounding. Mendelian randomization, a novel research approach, leverages publicly available data, improves research efficiency, avoids confounding bias, and employs randomized grouping similar to RCTs, providing additional evidence for causality.

Using Mendelian randomization (Wald ratio method) with metabolite-specific SNVs as instrumental variables, it could be found that serum lithocholate sulfate (1) was significantly and positively associated with chronic lymphocytic leukemia (CLL) risk (OR = 2.19, 95% CI: 1.45–3.31, *p* = 2 × 10^−4^). This finding suggests that altered lithocholate sulfate (1) levels or its metabolic pathway may contribute to CLL pathogenesis. Lithocholate sulfate (1) is a secondary bile acid generated from lithocholic acid via 7α-dehydroxylation by gut microbiota and can be desulfated back to lithocholic acid by microbial sulfatases. Lithocholic acid has immunomodulatory and cytotoxic effects, and sulfation serves as a detoxification mechanism, though the sulfated form retains biological activity. Accumulation of lithocholic acid and its sulfate can promote carcinogenesis by generating reactive oxygen species, forming DNA adducts, and inhibiting DNA repair enzymes [27,28,29]. Notably, several drugs, including abiraterone, ibrexafungerp, methyldopa, palbociclib, pindolol, prasterone, tamoxifen, and terbutaline, are reported substrates of lithocholate sulfate (1) metabolism. Avoidance of these medications could hypothetically reduce CLL risk and represents a potential direction for future clinical intervention.

Based on the Mendelian randomization findings, four mechanistic hypotheses can be proposed:

Firstly, genetically determined increases in lithocholate sulfate (1) (LCA-S) can be interpreted as a systemic readout of enhanced LCA to LCA-S detoxification flux. Lithocholic acid (LCA) itself is genotoxic, capable of inhibiting DNA polymerase β, forming DNA adducts, and inducing oxidative stress, thereby increasing genomic instability and opportunities for aberrant B-cell clone selection. It is therefore hypothesized that, in the peripheral B-cell niche, high LCA flux elevates the probability of generating or retaining abnormal clones, promoting CLL susceptibility. Testable predictions include positive correlations between plasma LCA-S levels and 8-oxoguanine content, base-excision repair/polymerase-β activity, DNA adduct burden, and oxidative stress markers in peripheral B cells [29,30].

Secondly, bile acid receptor signaling (TGR5/FXR/VDR) may skew the immune microenvironment toward immunosuppression. Bile acids act not only as metabolic intermediates but also as immunomodulatory ligands that regulate dendritic cell activation, macrophage polarization, and T-cell differentiation through TGR5, FXR, and VDR signaling. Isoallo-LCA, for example, has been shown to promote Treg differentiation. An LCA/LCA-S-enriched bile acid pool may bias the peripheral immune microenvironment toward an immunosuppressive phenotype, thereby supporting B-cell clonal persistence. Testable predictions include a positive association between LCA-S and peripheral Treg/Tfh ratios or receptor-specific transcriptional signatures, as well as causal shifts in immune phenotypes and B-cell activity upon pharmacological agonism or antagonism of TGR5/FXR/VDR [31,32,33].

Thirdly, limited sulfation capacity (SULT2A1) and drug competition could amplify upstream LCA burden. Human SULT2A1 is the key enzyme mediating LCA sulfation. Genetic variants or pharmacological inhibitors that constrain its capacity may lead to concurrent elevation of unconjugated LCA and abnormal accumulation of LCA-S, amplifying genotoxic and immune-modulatory effects. Experimental evidence shows that tamoxifen/endoxifen can inhibit hSULT2A1 activity; abiraterone undergoes major sulfate conjugation (abiraterone sulfate and N-oxide abiraterone sulfate) via SULT2A1; and terbutaline undergoes extensive sulfation in humans. Testable prediction: in individuals carrying low-activity SULT2A1 alleles or receiving these medications, the association between LCA-S levels and CLL susceptibility/biomarkers will be stronger [34,35].

Finally, microbiota-mediated 7α-dehydroxylation may enhance LCA production, coupling barrier function and systemic exposure. LCA is primarily generated by gut microbiota through 7α-dehydroxylation of primary bile acids, one of the most critical bile acid transformations in the colon. Microbiota composition and host FXR/TGR5 signaling jointly regulate intestinal barrier integrity and systemic bile acid exposure. It could hypothesize that enrichment of LCA-producing taxa or hyperactivation of this pathway, combined with barrier or receptor dysregulation, leads to elevated circulating bile acids (including LCA-S) and perturbs peripheral immune homeostasis, thereby increasing CLL susceptibility. Testable predictions include concordant increases in fecal bile acid profiles, 7α-dehydroxylation gene abundance, and plasma LCA-S; microbial or pharmacological interventions that dampen bile acid signaling should reduce peripheral immunosuppressive phenotypes [36,37].

This study also identified thyroxine as a protective factor for acute myeloid leukemia (AML), suggesting that exposure to thyroxine inhibitors may increase AML risk. Based on the present findings, eltrombopag, valproic acid, and chlorambucil are recognized thyroxine inhibitors, and their clinical use may be associated with leukemogenic potential. Notably, published studies indicate that the mechanisms linking these agents to leukemogenesis or leukemia-related biological effects differ.

In high-risk MDS defined by the International Prognostic Scoring System intermediate-2/high category with thrombocytopenia, adding eltrombopag to azacitidine did not improve the primary endpoint of platelet transfusion-free status during cycles 1 to 4 (16% vs. 31%), and the phase III trial was stopped early for futility and safety reasons. Progression to acute myeloid leukemia occurred more often with eltrombopag (18% vs. 11%), and overall survival showed a non-significant trend toward harm (hazard ratio 1.42; 95% CI 0.97–2.08) [38].

Valproic acid, a histone deacetylase inhibitor (HDACi), has been extensively studied in AML for its epigenetic effects, including induction of leukemic cell differentiation, apoptosis, and cell-cycle arrest. Mechanistically, valproic acid inhibits HDAC activity, increases histone acetylation, opens chromatin structure, and restores the expression of tumor suppressor genes, such as differentiation-related transcription factors, thereby exerting antileukemic effects [39,40,41]. However, in vitro studies have also shown that while valproic acid induces differentiation of AML progenitor cells, it may paradoxically enhance leukemia stem cell self-renewal in some cases, potentially contributing to disease persistence or relapse [42,43].

Chlorambucil, an alkylating chemotherapeutic agent, is widely used in the treatment of CLL, Hodgkin lymphoma, and ovarian cancer. Its genotoxicity and the associated risk of secondary leukemia, particularly therapy-related AML (t-AML), are well recognized. Mechanistically, chlorambucil generates ethyleniminium ion intermediates that form crosslinks at the N7 position of guanine, resulting in DNA double-strand breaks. While this mechanism is cytotoxic to malignant cells, it may also damage normal hematopoietic stem and progenitor cells, thereby drive clonal evolution and increase the risk of leukemogenesis [44,45,46].

The drug-matching results from this study suggest a potential interaction between thyroxine levels, drug exposure, and AML risk. However, the pathogenic pathways implicated by each drug are heterogeneous. At this stage, these findings should be regarded as hypothesis-generating signals and potential stratification markers rather than clinical contraindications. Future work should integrate pharmacoepidemiological studies, thyroxine-related biomarkers, and mechanistic experiments to validate these associations in a stratified manner.

Despite the significant finding, this study has several limitations. Firstly, under a single-sentinel Wald-ratio design based on summary-level data, we could not apply multi-variant heterogeneity or pleiotropy diagnostics. Secondly, this study was restricted to participants of predominantly European genetic ancestry, so extrapolation to other populations requires confirmation in more diverse cohorts. Moreover, Mendelian-randomization estimates reflect lifelong, genetically proxied differences rather than short-term pharmacologic modulation, meaning translation across exposure periods or timings is not direct.

## 5. Conclusions

In this two-sample Mendelian randomization analysis linking 83 plasma metabolites to four leukemia subtypes, we identified a single metabolite with significance, lithocholate sulfate (1) for chronic lymphocytic leukemia, and a broader set of suggestive associations that warrant follow-up. These findings prioritize specific metabolic pathways for mechanistic investigation and, when mapped to existing pharmacology, generate tractable hypotheses about drug–metabolite–gene interactions relevant to leukemogenesis. At the same time, the estimates should be interpreted as effects of lifelong, genetically proxied exposures rather than short-term pharmacologic modulation.

Methodologically, the single-sentinel strategy increased interpretability and reduced redundancy, and harmonization alongside a catalog-based pleiotropy screen limited obvious alternative pathways. This sentinel instrumental variable selection strategy enhances the representativeness, interpretability, and therapeutic relevance of the analysis.

Overall, this study provides a transparent, reproducible screen of metabolite–leukemia relationships, highlights a small number of convergent pathways, and lays out a practical agenda for validation and mechanism, moving from correlation toward causally informed biology. Future work should replicate prioritized signals in independent and ancestry-diverse datasets; evaluate directionality and shared causal variants using Steiger tests and colocalization; and probe mechanism through integration with expression and splicing QTLs, fine-mapping, and experimental perturbation. Pharmaco-epidemiologic analyses can test predicted drug–metabolite–outcome interactions, and multivariable or network MR may help separate correlated metabolites within shared pathways. Together, these steps will clarify whether the observed associations reflect pathway-specific causal effects and will define their translational value for risk stratification or prevention.

## Figures and Tables

**Table 1:** DrugBank-annotated clinical drugs targeting effector gene SULT2A1 for the CLL-significant metabolite lithocholate sulfate (1).

Metabolite or Metabolite Ratios	Effector Genes	Protein Type	DrugBank_ID	Drug_Name	Relation	Antagonist	Agonist	Substrate	Inhibitor	Inducer
lithocholate sulfate (1)	SULT2A1	Enzyme	DB05812	Abiraterone	EnzymeBond	0	0	1	0	0
lithocholate sulfate (1)	SULT2A1	Enzyme	DB12471	Ibrexafungerp	EnzymeBond	0	0	1	0	0
lithocholate sulfate (1)	SULT2A1	Enzyme	DB00968	Methyldopa	EnzymeBond	0	0	1	0	0
lithocholate sulfate (1)	SULT2A1	Enzyme	DB09073	Palbociclib	EnzymeBond	0	0	1	0	0
lithocholate sulfate (1)	SULT2A1	Enzyme	DB00960	Pindolol	EnzymeBond	0	0	1	0	0
lithocholate sulfate (1)	SULT2A1	Enzyme	DB01708	Prasterone	EnzymeBond	0	0	1	0	0
lithocholate sulfate (1)	SULT2A1	Enzyme	DB00675	Tamoxifen	EnzymeBond	0	0	1	0	0
lithocholate sulfate (1)	SULT2A1	Enzyme	DB00871	Terbutaline	EnzymeBond	0	0	1	0	0

Note: The table links the Mendelian-randomization significant metabolite for CLL, lithocholate sulfate (1) to its assigned effector gene SULT2A1 and lists clinical drugs recorded in DrugBank as interacting with this enzyme. All listed agents (abiraterone, ibrexafungerp, methyldopa, palbociclib, pindolol, prasterone, tamoxifen, terbutaline) are annotated with Relation: EnzymeBond; Substrate: yes; Antagonist: no; Agonist: no; Inhibitor: no; Inducer: no. Binary role indicators use 1 for yes and 0 for no; DrugBank_ID and Drug_Name correspond to the DrugBank reference entry. CLL, chronic lymphocytic leukemia; SULT2A1, sulfotransferase family 2A member 1.

## Data Availability

The data presented in this study are available in article and Supplementary Material. Public summary statistics for leukemia outcomes (AML, CML, ALL, CLL) were obtained from the FinnGen R12 resource and the UK Biobank-based meta-analysis. Plasma metabolite summary statistics were taken from the Canadian Longitudinal Study on Aging (CLSA) cohort accessed via the GWAS Catalog (European ancestry: GCST90199621–GCST90201020). Variant cross-references were retrieved from the GWAS Catalog, and pharmacologic annotations were sourced from DrugBank.

## Supplementary Materials

The following supporting information can be downloaded at: https://www.mdpi.com/article/10.3390/metabo15110719/s1, Supplementary Table S1. GWAS data sources and cohort characteristics for plasma metabolite and leukemia; Supplementary Table S2. GWAS Catalog cross-references for instrument SNVs; Supplementary Table S3. Characteristics of genetic instruments for circulating metabolites with 83 sentinel SNVs (across AML, CML, ALL, and CLL); Supplementary Table S4. R^2^ and F statistics for sentinel variants of metabolite instruments; Supplementary Table S5. Metabolite–leukemia MR associations across subtypes with sentinel details (suggestive significance); Supplementary Table S6. Single-sentinel Mendelian randomization estimates for AML; Supplementary Table S7. Single-sentinel Mendelian randomization estimates for CML; Supplementary Table S8. Single-sentinel Mendelian randomization estimates for ALL; Supplementary Table S9. Single-sentinel Mendelian randomization estimates for CLL; Supplementary Table S10. The power for AML at prespecified odds ratios; Supplementary Table S11. The power for CML at prespecified odds ratios; Supplementary Table S12. The power for ALL at prespecified odds ratios; Supplementary Table S13. The power for CLL at prespecified odds ratios; Supplementary Table S14. DrugBank-mapped drugs targeting effector genes of metabolites with suggestive significance; Supplementary Table S15. STROBE-MR checklist of recommended items to address in reports of Mendelian randomization studies; Supplementary Data: Supplementary Tables S1–S15.

## Abbreviations

ALL, acute lymphoblastic leukemia; AML, acute myeloid leukemia; CD36, cluster of differentiation 36; CDK4/6, cyclin-dependent kinase 4/6; CI, confidence interval; CLL, chronic lymphocytic leukemia; CML, chronic myeloid leukemia; CRISPR, clustered regularly interspaced short palindromic repeats; DHODH, dihydroorotate dehydrogenase; DNA, deoxyribonucleic acid; dNTP, deoxyribonucleoside triphosphate; FAO, fatty-acid oxidation; FinnGen, Finnish biobank research project; FXR, farnesoid X receptor; GWAS, genome-wide association study; HDACi, histone deacetylase inhibitor; IDH, isocitrate dehydrogenase; IV, instrumental variable; LCA, lithocholic acid; LCA-S, lithocholate sulfate (1); LD, linkage disequilibrium; LSC, leukemia stem cell; MDS, myelodysplastic syndrome; MLL, mixed-lineage leukemia; MR, Mendelian randomization; MTHFD2, methylenetetrahydrofolate dehydrogenase 2; MYC, MYC proto-oncogene; OR, odds ratio; OXPHOS, oxidative phosphorylation; QTL, quantitative trait locus; RCT, randomized controlled trial; SD, standard deviation; SNV, single-nucleotide variant; S-phase, DNA synthesis phase of the cell cycle; SULT2A1, sulfotransferase family 2A member 1; STROBE-MR, Strengthening the Reporting of Observational Studies in Epidemiology using Mendelian Randomization; TET2, ten-eleven translocation methylcytosine dioxygenase 2; Tfh, follicular helper T cell; TGR5, G protein-coupled bile acid receptor 1; Treg, regulatory T cell; VDR, vitamin D receptor; VPA, valproic acid.
