# Supplementary material for: Causal Effects of Plasma Metabolites on Leukemia: A Mendelian Randomization Study
Source: Metabolites. 2025 Nov 3;15(11):719. doi: 10.3390/metabo15110719 (PMC12654070; doi:10.3390/metabo15110719)
Supplement: Supplementary file 1 [file metabolites-15-00719-s001.zip › Supplementary Table S15_STROBE-MR-checklist_metabolites-3940177.pdf]

Supplementary Table S15. STROBE-MR checklist of recommended items to address in reports of Mendelian randomization studies [1, 2]

Item No.	Section	Checklist item	Page No.	Relevant text from manuscript
1	**TITLE and ABSTRACT**	Indicate Mendelian randomization (MR) as the study's design in the title and/or the abstract if that is a main purpose of the study	1	Title: "Causal Effects of Plasma Metabolites on Leukemia: A Mendelian Randomization Study"<br><br>Abstract: "A two-sample Mendelian randomization (MR) using summary-level genome-wide association study statistics was conducted."
	**INTRODUCTION**			
2	**Background**	Explain the scientific background and rationale for the reported study. What is the exposure? Is a potential causal relationship between exposure and outcome plausible? Justify why MR is a helpful method to address the study question	2	"Metabolic dysregulation is a hallmark of cancer and affects lineage commitment, genome maintenance, and tumor–immune interactions. Work in myeloid malignancies illustrates how metabolite changes can act as proximal drivers of leukemogenesis. Mutant IDH1 and IDH2 generate 2-hydroxyglutarate, disrupt TET2 function, enforce DNA hypermethylation, and impair differentiation in acute myeloid leukemia, showing that metabolic alterations can be causal rather than merely correlative."<br>"Conventional observational studies have long reported metabolic abnormalities in leukemia across amino acid, lipid, and organic-acid pathways, but such evidence is vulnerable to confounding and reverse causation. Cross-sectional or cohort correlations cannot by themselves establish whether metabolic changes are causes of disease or consequences of it. Mendelian randomization uses germline variants strongly associated with the exposure, namely plasma metabolite levels, as instrumental variables. Under its core assumptions, Mendelian randomization (MR) reduces confounding and mitigates reverse causation bias, providing complementary causal evidence to randomized trials."

3	**Objectives**	State specific objectives clearly, including pre-specified causal hypotheses (if any). State that MR is a method that, under specific assumptions, intends to estimate causal effects	2	“Collectively, these frameworks underscore that subtype-specific etiology is the biologically appropriate unit for epidemiology and translational research, and they motivate analyses that respect recognized genomic and clinical heterogeneity”<br>“Despite progress, an important knowledge gap remains: while metabolite genetics has been mapped broadly and MR has implicated metabolites in diverse complex traits, the causal relevance of specific plasma metabolites for leukemia subtypes has not been systematically evaluated at scale using a harmonized, single-sentinel framework”
	**METHODS**			
4	**Study design and data sources**	Present key elements of the study design early in the article. Consider including a table listing sources of data for all phases of the study. For each data source contributing to the analysis, describe the following:		
	a)	Setting: Describe the study design and the underlying population, if possible. Describe the setting, locations, and relevant dates, including periods of recruitment, exposure, follow-up, and data collection, when available.	2	“A two-sample Mendelian randomization was conducted to evaluate the causal effects of human plasma metabolites on leukemia. Plasma metabolite summary statistics came from the Canadian Longitudinal Study on Aging (CLSA) cohort with data deposited in the GWAS Catalog under accession ranges GCST90199621–GCST90201020 for European ancestry. Summary statistics for leukemia were taken from the FinnGen R12 and UK Biobank meta-analysis resource.”
	b)	Participants: Give the eligibility criteria, and the sources and methods of selection of participants. Report the sample size, and whether any power or sample size calculations were carried out prior to the main analysis	2,5	“The GWAS for metabolites and their ratios included 8299 individuals.”<br>“The case and control counts were as follows: acute myeloid leukemia 731 and 793,587; chronic myeloid leukemia 474 and 793,588; acute lymphoblastic leukemia 313 and 706,277; chronic lymphocytic leukemia 1,585 and 793,582.”

			“We also summarized post-hoc power at prespecified odds-ratio effect sizes of 0.90, 1.20, and 1.50 while accounting for the case–control ratio of each outcome.”
c)	Describe measurement, quality control and selection of genetic variants	4	“Instrumental variables were defined using a single-sentinel strategy for the exposure. In this study, the sentinel was defined as the SNV showing the strongest association with the metabolite that had the largest variance explained (R^2) among the independent genome-wide significant ($P < 5 \times 10^{-8}$) SNPs assigned to effector genes. To minimize pleiotropic effects, we excluded the FADS gene region, which is associated with multiple metabolites, as well as the major histocompatibility complex region on chromosome 6. This sentinel instrumental variable selection strategy enhances the representativeness, interpretability, and therapeutic relevance of the analysis. For metabolites with multiple independent signals, only the top sentinel was retained for the primary analysis, yielding a one-to-one mapping of metabolites to instruments. The final instrument list comprised 83 unique metabolites and 83 corresponding sentinels.”<br><br>“When a sentinel was missing in an outcome file, an LD proxy was permitted un-der a predefined rule. Proxies were searched within a matched-ancestry reference with $r^2 > 0.8$; when multiple candidates were available, the proxy with the highest r^2 was selected, and the proxy indicator with its r^2 value was carried forward into the analysis dataset. Effect alleles and allele frequencies were then harmonized between exposure and outcome, including sign changes when required by strand orientation.”
d)	For each exposure, outcome, and other relevant variables, describe methods of assessment and diagnostic criteria for diseases	3	“Each metabolite was treated as an exposure and each leukemia subtype as a separate outcome. Because clinical and genetic architectures differ by subtype, all analyses were performed independently for each outcome

					and results were reported in parallel. Exposure and outcome ancestries were aligned as closely as possible according to the source studies. Outcome effects are on the log-odds scale and are presented as odds ratios after exponentiation for interpretability.”
		e)	Provide details of ethics committee approval and participant informed consent, if relevant	N/A	
5	**Assumptions**		Explicitly state the three core IV assumptions for the main analysis (relevance, independence and exclusion restriction) as well assumptions for any additional or sensitivity analysis	3 and<br>Figure 1	“The single-nucleotide variations (SNVs) used to demonstrate causal effects in the MR analysis must satisfy three key assumptions: Firstly, the IVs must be closely related to the exposure; Secondly, the IVs are not related to any confounders of the risk factor-outcome association; Thirdly, the IVs do not affect the outcome through any pathway other than the exposure of interest. The fundamental assumptions of MR are depicted in Figure 1.”
6	**Statistical methods: main analysis**		Describe statistical methods and statistics used		
		a)	Describe how quantitative variables were handled in the analyses (i.e., scale, units, model)	3-5	“For binary outcomes we report the odds ratio per one standard-deviation higher genetically predicted metabolite level together with ninety-five percent confidence intervals.”<br><br>“Outcome effects are on the log-odds scale and are presented as odds ratios after exponentiation for interpretability.”
		b)	Describe how genetic variants were handled in the analyses and, if applicable, how their weights were selected	4	**“**Instrumental variables were defined using a single-sentinel strategy for the exposure. In this study, the sentinel was defined as the SNV showing the strongest association with the metabolite that had the largest variance explained (R^2) among the independent genome-wide significant ($P < 5 \times 10^{-8}$) SNPs assigned to effector genes. To minimize pleiotropic effects, we excluded the FADS gene region, which is associated with multiple metabolites, as well as the major histocompatibility complex region on chromosome 6. This sentinel instrumental

				variable selection strategy enhances the representativeness, interpretability, and therapeutic relevance of the analysis. For metabolites with multiple independent signals, only the top sentinel was retained for the primary analysis, yielding a one-to-one mapping of metabolites to instruments. The final instrument list comprised 83 unique metabolites and 83 corresponding sentinels."<br><br>"When a sentinel was missing in an outcome file, an LD proxy was permitted under a predefined rule. Proxies were searched within a matched-ancestry reference with $r^2 > 0.8$; when multiple candidates were available, the proxy with the highest r^2 was selected, and the proxy indicator with its r^2 value was carried forward into the analysis dataset."
		c) Describe the MR estimator (e.g. two-stage least squares, Wald ratio) and related statistics. Detail the included covariates and, in case of two-sample MR, whether the same covariate set was used for adjustment in the two samples	4	"Because each metabolite contributed a single instrument, the primary estimator was the Wald ratio …"
		d) Explain how missing data were addressed	4	When a sentinel was missing in an outcome file, an LD proxy was permitted under a predefined rule.
		e) If applicable, indicate how multiple testing was addressed	5	"For multiplicity control all P values are two-sided, and our primary decision rule is Bonferroni within outcome. With eighty-three metabolites per outcome the significance threshold equals 0.05 divided by eighty-three, which is 6.02×10^{-4}."
7	**Assessment of assumptions**	Describe any methods or prior knowledge used to assess the assumptions or justify their validity	5	"To quantify instrument strength, we computed the variance explained (R^2) for each sentinel from the exposure-side effect size and allele frequency, and then derived the first-stage F statistic… These metrics accompany the MR estimates and were not used for additional filtering beyond the exposure-side genome-wide significance threshold. We also summarized post-hoc power at prespecified odds-ratio effect sizes of 0.90, 1.20, and 1.50 while accounting for the case–control ratio of each outcome. To limit horizontal pleiotropy, we performed a programmatic screen of every sentinel or selected proxy against the GWAS Catalog…

				Variants flagged by this screen were compiled into a drop list, and the corresponding rsIDs were removed from the MR input… We retained the catalog annotations for the remaining instruments to aid interpretation in the results.”
8	**Sensitivity analyses and additional analyses**	Describe any sensitivity analyses or additional analyses performed (e.g. comparison of effect estimates from different approaches, independent replication, bias analytic techniques, validation of instruments, simulations)	5	“We also summarized post-hoc power at prespecified odds-ratio effect sizes of 0.90, 1.20, and 1.50 while accounting for the case–control ratio of each outcome.”<br><br>“For multiplicity control all P values are two-sided, and our primary decision rule is Bonferroni within outcome. With eighty-three metabolites per outcome the significance threshold equals 0.05 divided by eighty-three, which is 6.02×10^{-4}.”
9	**Software and pre-registration**			
	a)	Name statistical software and package(s), including version and settings used	5	“All analyses were conducted in R… The core packages were TwoSampleMR… and MendelianRandomization… and data.table, dplyr, tidyr or reshape2, ggplot2, forestplot, plotly, htmlwidgets, and webshot…”<br><br>“Data were analyzed using the TwoSampleMR (version 0.6.22) and Mendelian Randomization (0.10.0) packages in the statistical program R (version 4.5.1; the R Foundation for Statistical Computing).”
	b)	State whether the study protocol and details were pre-registered (as well as when and where)	N/A	
	**RESULTS**			
10	**Descriptive data**			
	a)	Report the numbers of individuals at each stage of included studies and reasons for exclusion. Consider use of a flow diagram	N/A	
	b)	Report summary statistics for phenotypic exposure(s), outcome(s), and other	N/A	

			relevant variables (e.g. means, SDs, proportions)		
		c)	If the data sources include meta-analyses of previous studies, provide the assessments of heterogeneity across these studies	N/A	
		d)	For two-sample MR:<br>i. Provide justification of the similarity of the genetic variant-exposure associations between the exposure and outcome samples<br>ii. Provide information on the number of individuals who overlap between the exposure and outcome studies	N/A	
11	**Main results**				
		a)	Report the associations between genetic variant and exposure, and between genetic variant and outcome, preferably on an interpretable scale	Supplementary Table 3	
		b)	Report MR estimates of the relationship between exposure and outcome, and the measures of uncertainty from the MR analysis, on an interpretable scale, such as odds ratio or relative risk per SD difference	Supplementary Table 5	
		c)	If relevant, consider translating estimates of relative risk into absolute risk for a meaningful time period	N/A	
		d)	Consider plots to visualize results (e.g. forest plot, scatterplot of associations between genetic variants and outcome versus between genetic variants and exposure)	Figure 2-4	
12	**Assessment of assumptions**				
		a)	Report the assessment of the validity of the assumptions	N/A	
		b)	Report any additional statistics (e.g., assessments of heterogeneity across genetic variants, such as I^2, Q statistic or E-value)	N/A	
13	**Sensitivity analyses and additional analyses**				
		a)	Report any sensitivity analyses to assess the robustness of the main results to violations of the assumptions	6, Supplementary Table 4	"R^2 and F statistics for each instrument are reported in Supplementary Table 4. Consistent with the conventional MR rule that instrument strength should exceed F greater than 10, all sentinel instruments met this requirement; after ranking by F, the smallest value was 36.5 and the largest was 4013, indicating high overall

					instrument quality (Supplementary Table 4)."
		b)	Report results from other sensitivity analyses or additional analyses	6, Supplementary Table 10–13	"To aid interpretation of null and suggestive findings, statistical power was evaluated at prespecified odds ratios of 0.90, 1.20, and 1.50 for each leukemia subtype; subtype-specific summaries are provided in Supplementary Table 10-13."
		c)	Report any assessment of direction of causal relationship (e.g., bidirectional MR)	N/A	
		d)	When relevant, report and compare with estimates from non-MR analyses	N/A	
		e)	Consider additional plots to visualize results (e.g., leave-one-out analyses)	N/A	
	**DISCUSSION**				
14	**Key results**		Summarize key results with reference to study objectives	11,12	**"**Using a uniform MR framework, we tested whether genetically proxied metabolite levels influence the risks of AML, CML, ALL, and CLL.**"**<br>"Using Mendelian randomization (Wald ratio method) with metabolite-specific SNVs as instrumental variables, it could be found that serum lithocholate sulfate (1) was significantly and positively associated with chronic lymphocytic leukemia (CLL) risk (OR = 2.19, 95% CI: 1.45–3.31, P = 2×10^{-4})."
15	**Limitations**		Discuss limitations of the study, taking into account the validity of the IV assumptions, other sources of potential bias, and imprecision. Discuss both direction and magnitude of any potential bias and any efforts to address them	14	"Despite the significant finding, this study has several limitations. Firstly, under a single-sentinel Wald-ratio design based on summary-level data, we could not apply multivariant heterogeneity or pleiotropy diagnostics. Secondly, this study was restricted to participants of predominantly European genetic ancestry, so extrapolation to other populations requires confirmation in more diverse cohorts.

					Moreover, Mendelian-randomization estimates reflect lifelong, genetically proxied differences rather than short-term pharmacologic modulation, meaning translation across exposure periods or timings is not direct.”
16	**Interpretation**				
		a)	Meaning: Give a cautious overall interpretation of results in the context of their limitations and in comparison with other studies	N/A	
		b)	Mechanism: Discuss underlying biological mechanisms that could drive a potential causal relationship between the investigated exposure and the outcome, and whether the gene-environment equivalence assumption is reasonable. Use causal language carefully, clarifying that IV estimates may provide causal effects only under certain assumptions	12	**“**Lithocholate sulfate (1) is a secondary bile acid generated from lithocholic acid via 7α-dehydroxylation by gut microbiota and can be desulfated back to lithocholic acid by microbial sulfatases. Lithocholic acid has immunomodulatory and cytotoxic effects, and sulfation serves as a detoxification mechanism, though the sulfated form retains biological activity. Accumulation of lithocholic acid and its sulfate can promote carcinogenesis by generating reactive oxygen species, forming DNA adducts, and inhibiting DNA repair enzymes.**”**
		c)	Clinical relevance: Discuss whether the results have clinical or public policy relevance, and to what extent they inform effect sizes of possible interventions	12.13	“Notably, several drugs—including abiraterone, ibrexafungerp, methyldopa, palbociclib, pindolol, prasterone, tamoxifen, and terbutaline, are reported substrates of lithocholate sulfate (1) metabolism. Avoidance of these medications could hypothetically reduce CLL risk and represents a potential direction for future clinical intervention.”
17	**Generalizability**		Discuss the generalizability of the study results (a) to other populations, (b) across other exposure periods/timings, and (c) across other levels of exposure	14	“Secondly, this study was restricted to participants of predominantly European genetic ancestry, so extrapolation to other populations requires confirmation in more diverse cohorts. Moreover, Mendelian-randomization estimates reflect lifelong, genetically proxied differences rather than short-term pharmacologic modulation, meaning translation across exposure periods or timings is not direct.”

	**OTHER INFORMATION**			
18	**Funding**	Describe sources of funding and the role of funders in the present study and, if applicable, sources of funding for the databases and original study or studies on which the present study is based	15	See Funding section
19	**Data and data sharing**	Provide the data used to perform all analyses or report where and how the data can be accessed, and reference these sources in the article. Provide the statistical code needed to reproduce the results in the article, or report whether the code is publicly accessible and if so, where	Supplementary Table 5	
20	**Conflicts of Interest**	All authors should declare all potential conflicts of interest	16	"The authors declare no competing interests."

This checklist is copyrighted by the Equator Network under the Creative Commons Attribution 3.0 Unported (CC BY 3.0) license.

1. Skrivankova, V.W.; Richmond, R.C.; Woolf, B.A.R.; Yarmolinsky, J.; Davies, N.M.; Swanson, S.A.; VanderWeele, T.J.; Higgins, J.P.T.; Timpson, N.J.; Dimou, N.; et al. Strengthening the Reporting of Observational Studies in Epidemiology Using Mendelian Randomization: The STROBE-MR Statement. *JAMA* 2021, 326, 1614. https://doi.org/10.1001/jama.2021.18236

2. Skrivankova, V.W.; Richmond, R.C.; Woolf, B.A.R.; Davies, N.M.; Swanson, S.A.; VanderWeele, T.J.; et al. Strengthening the Reporting of Observational Studies in Epidemiology using Mendelian Randomisation (STROBE-MR): Explanation and Elaboration. *BMJ* 2021;375:n2233. https://doi.org/10.1136/bmj.n2233
